# Determining factors influencing hospital stay for individuals admitted with diabetes-related ketoacidosis – findings from DEKODE length of stay quality improvement project

**DOI:** 10.1016/j.clinme.2024.100255

**Published:** 2024-10-19

**Authors:** Ankita Gupta, Benedict Brazier, Lakshmi Rengarajan, Parth Narendran, Punith Kempegowda, DEKODE Working Group

**Affiliations:** a Queen Elizabeth Hospital, Birmingham, United Kingdom; b Institute of Immunology and Immunotherapy, University of Birmingham, Birmingham, United Kingdom; c Institute of Applied Health Research, University of Birmingham, Birmingham, United Kingdom

**Keywords:** Diabetic ketoacidosis, Length of stay

## Abstract

There are significant variations in discharge post-diabetes-related ketoacidosis (DKA) hospitalisation, yet there is a paucity of research to understand or minimise the reasons. This quality improvement project (QIP) aimed to identify reasons for post-DKA discharge delays and assess intervention efficacy. Utilising the Digital Evaluation of Ketosis and Other Diabetes-related Emergencies (DEKODE) model, data from 177 DKA episodes from January 2021 to September 2023 across three hospitals were analysed. Factors affecting discharge were investigated through a plan, do, study, act (PDSA) methodology. While interventions focused on optimising data collection and refining discharge guidelines, no significant reduction in DKA duration or length of stay was observed. Findings highlight post-DKA hospitalisation's multifaceted nature and the limited impact of simple interventions. Collaborative efforts and further research are necessary to develop effective strategies for expedited discharge and improved patient care. This study's comprehensive tracking and analysis tool offers valuable insights for future interventions in managing DKA-related hospitalisations.

## Introduction

Diabetes-related ketoacidosis (DKA) is one of the most common acute emergencies in people with diabetes, leading to unplanned hospitalisation.^1^ The most common precipitating factors for DKA include intercurrent illness and suboptimal adherence to insulin therapy,^2^^,^^3^ and its management includes intravenous rehydration and insulin administration to stop ketosis.^4^^,^^5^ While some studies have tried to identify factors influencing hospital stay post-DKA resolution,^6^^,^^7^ they did not explore how these factors can ensure faster and safer discharge. The Joint British Diabetes Societies for Inpatient Care (JBDS-IP) has a guideline on discharging diabetes patients safely; however, this is not DKA-specific.^8^

In this quality improvement project (QIP), we investigated the reasons behind delays in discharging patients after experiencing DKA and assessed the effectiveness of interventions to reduce these delays. Our goal was to explore whether simple interventions can efficiently minimise delays in post-DKA discharge. By continuously collecting data, we aimed to evaluate the ongoing impact of these interventions over time. Towards this, we set three objectives:

1. To identify factors influencing time from DKA resolution until the person becomes medically fit for discharge (MFFD).
2. To identify factors that influence the time from MFFD to the time of discharge.
3. To identify trends in DKA outcomes (DKA duration and length of stay) following interventions that can reduce the length of stay post-DKA.

## Methods

The Digital Evaluation of Ketosis and Diabetes-related Emergencies (DEKODE) model was employed in developing this QIP. The model systematically gathers comprehensive data about the management of DKA from initial diagnosis to resolution.^9^ We included all DKA episodes from January 2021 until September 2023 across three hospitals within the University Hospitals Birmingham NHS Foundation Trust (UHB), UK. DKA was diagnosed as per JBDS-IP criteria (blood glucose exceeding 11 mmol/L or the individual has a history of diabetes, pH level of 7.3 or lower or bicarbonate levels of 15 mmol/L or lower, and ketone levels of 3 mmol/L or higher).^4^

Individual patient episodes were meticulously reviewed through electronic patient records for various data associated with DKA admission. Reasons for hospital stay after DKA resolution until the individual was declared medically fit for discharge (MFFD) and after MFFD until discharge was documented. We also collected data on the date and time when the individual with DKA was reviewed by a member of the diabetes team (comprising diabetes physicians and/or diabetic specialist nurses). The Charlson Comorbidity Index (CCI), which predicts 10-year survival in those with multiple health conditions, was also collected for each DKA episode. Incomplete data entries were excluded from subsequent analyses.

**Fig. 1:**
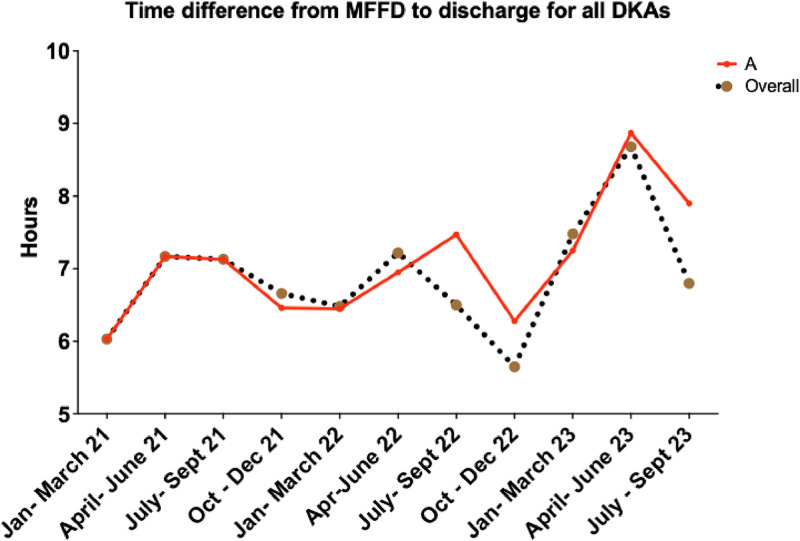
Time difference between MFFD and discharge for all DKA episodes in Hospital A. Graph showing overall time difference from MFFD (medically fit for discharge) at Hospital A over the data collection time period, and the overall median time across all three hospitals.

**Fig. 2:**
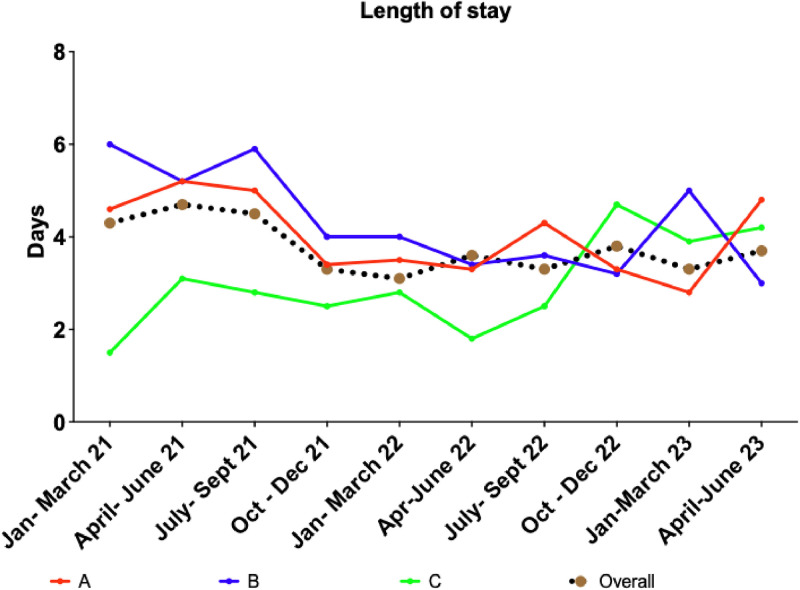
Graph of overall length of stay for all DKA episodes. Graph showing overall length of stay at Hospitals A, B and C over the data collection time period, and the overall median length of stay across all three hospitals.

We employed a plan, do, study, act (PDSA) methodology in three periods: period A (January– March 2023), period B (April–June 2023) and period C (July–September 2023), using the SQUIRE guidelines as a framework (Supplementary material 1). The overall goal was to reduce the time between a patient becoming medically fit for discharge (Fig. 1) and their actual time of discharge, which in turn would reduce the length of stay (Fig. 2).

### Period A (January–March 2023)

The data collection sheet on various aspects of medical care from DKA resolution until discharge was created through a consensus of experts based in hospitals included in the study (Supplementary material 2). Baseline data were collected retrospectively at the end of March 2023 for all DKA episodes between January 2021 and March 2023 by the study team. The first intervention at the end of March 2023 was a presentation of the findings with the diabetes team in Hospital A. Following their input, we revised the data collection tool to ensure a comprehensive collection of all relevant information (supplementary 1). A discharge delay was defined as a delay of more than 3 hours after being declared medically fit for discharge within regular working hours (Monday–Friday, 08:00–17:00, excluding bank holidays). Glycaemic control was further categorised as a cause of delay in achieving MFFD status, distinguishing between individuals on variable rate intravenous insulin infusion (VRIII) and those experiencing delays due to other reasons that influenced glycaemic control.

### Period B (April–June 2023)

The optimised data collection protocol was implemented in April 2023, and data were collected prospectively for all DKA episodes from that date onwards. We analysed data throughout this period to monitor changes in outcomes after sharing our findings with the diabetes team in Hospital A. We also used this period to ensure the ease of use of the optimised data collection protocol.

### Period C (July–September 2023)

At the end of June 2023, our second intervention was a presentation of our findings to the doctors working on the diabetes ward at Hospital A. These doctors ranged from foundation year 1 to internal medicine trainee year 2. We did this to enhance their awareness of the factors causing delays. One of the reasons highlighted was the repeat diabetes team referrals to advise on safe blood sugar levels before discharge. The study team prospectively collected data between July and September 2023. Our final intervention was a presentation to the diabetes team at Hospital A in October 2023. An agreement was reached to include the following statement in the diabetes team noting for all cases reviewed for DKA to facilitate faster discharge: ‘if the person with diabetes is clinically well, glucose is <20 mmol/L and ketones 0 mmol/L in two consecutive hours, then safe to discharge from a diabetes perspective’.

### Interventions

The first intervention centred on creating a consensus data collection tool. The second intervention included presenting our findings to the doctors working on the diabetes ward at Hospital A. The third intervention included creating the guidance statement clarifying discharge glucose and ketone targets, which we hope will reduce the total length of stay.

### Analysis

All episodes of DKA were analysed from 1 January 2021 at Hospital A, 21 October 2021 at Hospital B and 1 October 2022 at Hospital C. The variance in start dates between hospitals was due to a lack of access to appropriate clinical notes. Incomplete data were excluded, and the remainder were exported to Microsoft Excel.

The Shapiro–Wilk test was used to test for normality. Normally distributed data were described with means and standard deviations if continuous. Median and interquartile ranges were calculated for skewed data. Categorical variables were described with frequencies and percentages. Significance was assessed using the Kruskal–Wallis test for non-parametric variables and the chi-square test for categorical variables. Statistical significance was set at *p*<0.05.

### Ethical considerations

All data were anonymised with a DEKODE Group identifier number. The QIP was approved by the hospitals’ clinical governance team (CARMS-18745).

## Results

### Baseline characteristics of people admitted with DKA

A total of 177 episodes were included in the analyses. Shapiro–Wilk testing revealed a non-normal distribution for all outcomes. The median age was 41.0 (IQR 25.0–61.0) with a male preponderance (female:male ratio of 1:1.4) (Table 1). The three most common ethnicities in the DKA sample were White (61.0%), Asian (11.3%) and Black (8.4%). The median CCI of DKA episodes was significantly higher in those who died during admission (died vs discharged: 5 (21% estimated 10-year survival) vs 1 (96% estimated 10-year survival).

**Table 1:** Baseline characteristics of DKA episodes.

Baseline	Hospital A	Hospital B	Hospital C	Overall
	N = 72	N = 68	N = 37	N = 177
Age: median (interquartile range)	44.0 (30.8–59.8)	43.0 (22.0–61.3)	40.0 (20.0–55.0)	41.0 (25.0–61.0)
Female:male	1:1.4	1:1.3	1:1.5	1:1.4
Ethnicity (number of DKA episodes)				
White	54 (75.0%)	31 (45.6%)	23 (62.2%)	108 (61.0%)
Asian	6 (8.3%)	13 (19.1%)	1 (2.7%)	20 (11.3%)
Black	4 (5.6%)	6 (8.8%)	5 (13.5%)	15 (8.4%)
Unknown	3 (4.2%)	13 (19.1%)	7 (18.9%)	23 (13.0%)
Other	2 (2.7%)	2 (2.9%)	0 (0.0%)	4 (2.3%)
Mixed	3 (4.2%)	3 (4.5%)	1 (2.7%)	7 (4.0%)

Ethnicity: White includes British, Irish and other White backgrounds. Black includes Caribbean, African and other Black backgrounds. Asian includes Indian, Pakistani, Asian Bangladeshi, Chinese and other Asian backgrounds. Other includes other ethnic backgrounds.

### Factors influencing time from DKA resolution until the person becomes medically fit for discharge (MFFD)

Establishing glycaemic control before discharge, ongoing investigation and treatment, and diabetes specialist nurse (DSN) review were the three most common factors influencing stay from DKA resolution to MFFD across all hospitals. There was a significant difference in glycaemic control due to the use of VRII at hospital A between Jan–March 2023 vs Apr–June 2023 vs July–Sept 2023: 73.3% vs 25.0% vs 27.3% (*p*=0.00) and DSN review at hospital B between Jan–March 2023 vs Apr–June 2023 vs July–Sept 2023: 17.7% vs 61.5% vs 16.0% (*p*=0.001). There was no significant difference in ongoing investigations and treatments across all periods (Table 2).

**Table 2:** Reasons for hospital stay post-DKA resolution until MFFD.

Parameters	Hospital A N (%)			Sig.	Hospital B N (%)			Sig.	Hospital C N (%)			Sig.
	Q1	Q2	Q3		Q1	Q2	Q3		Q1	Q2	Q3	
DSN review	5 (16.7)	8 (40.0)	3 (13.6)	0.07	3 (17.7)	16 (61.5)	4 (16.0)	**0.001**	2 (16.7)	5 (38.5)	1 (8.3)	0.17
Glycaemic control (not MFFD)	3 (10.0)	7 (35.0)	7 (31.8)	0.07	3 (17.7)	7 (26.9)	7 (28.0)	0.72	3 (25.0)	3 (23.1)	5 (41.7)	0.54
Glycaemic control (on VRII)	22 (73.3)	5 (25.0)	6 (27.3)	**<0.01**	9 (52.9)	5 (19.2)	7 (28.0)	0.60	3 (25.0)	3 (23.1)	2 (16.7)	0.87
Ongoing Ix and Tx	7 (23.3)	10 (50.0)	10 (45.5)	0.11	7 (41.2)	12 (46.2)	16 (64.0)	0.27	5 (41.7)	5 (38.5)	7 (58.3)	0.57
Medical specialist review	3 (10.0)	6 (30.0)	3 (13.6)	0.16	3 (17.7)	5 (19.2)	4 (16.0)	0.96	0 (0.0)	3 (23.1)	2 (16.7)	0.22
Surgical specialist review	0 (0.0)	2 (10.0)	0 (0.0)	0.07	1 (5.9)	1 (3.9)	2 (8.0)	0.82	1 (8.3)	1 (7.7)	1 (8.3)	0.99
Psychiatrist review	1 (3.3)	3 (15.0)	1 (4.5)	0.25	0 (0.0)	0 (0.0)	0 (0.0)	n/a	0 (0.0)	1 (7.7)	0 (0.0)	0.60
Covid or needing O_2_	2 (6.7)	0 (0.0)	0 (0.0)	0.24	0 (0.0)	0 (0.0)	0 (0.0)	n/a	0 (0.0)	0 (0.0)	0 (0.0)	0.11
AHP review	0 (0.0)	0 (0.0)	0 (0.0)	n/a	0 (0.0)	0 (0.0)	0 (0.0)	n/a	0 (0.0)	1 (7.7)	1 (8.3)	0.60

Results are expressed in N (%) where appropriate. Significance (*p*-value), describing the comparison between JanuaryMarch 2023 (Q1) and April–June 2023 (Q2) and July–September 2023 (Q3) for each hospital. Significance set at *p*<0.05. *p*-values in bold are significant. AHP = allied health professional; DKA = diabetic ketoacidosis; DSN = diabetic specialist nurse; Ix = investigations; MFFD = medically fit for discharge; N = number; n/a = not applicable; O_2_ = oxygen; Sig.= *p*-value; Tx = treatments; VRII = variable rate insulin infusion.

There was no significant difference in the time from DKA diagnosis to diabetes doctor review across the periods in all three hospitals. However, there was a significant improvement in time from DKA diagnosis to DSN review (median duration in hours) in hospital B between Jan–March 2023 vs Apr–June 2023 vs July–Sept 2023: 40.1 vs 12.7 vs 16.4 (*p*=0.01). We did not notice a similar trend in hospitals A and C – Jan–March 2023 vs Apr–Jun 2023 vs July Sept 2023: Hospital A – 37.0 vs 20.5 vs 24.3 (*p*=0.15), Hospital C – 20.4 vs 24.6 vs 20.1 (*p*=0.24) (Table 2).

### Factors that influence the time from MFFD to the time of discharge

Once MFFD, the median time taken to be discharged was 7.1 vs 8.7 vs 7.8 hours (*p*=0.3) in Jan–Mar 2023 vs Apr–Jun 2023 vs July–Sept 2023. The most common reason for delay in discharge after becoming MFFD was awaiting discharge letters / TTOs (overall 9.1 hours; 7.5 hours for Hospital A, 8.2 hours for Hospital B, 8.0 hours for Hospital C) (Table 3).

**Table 3:** Reasons for hospital stay from MFFD till actual time of discharge.

Parameters	Hospital A N (%)			Sig.	Hospital B N (%)			Sig.	Hospital C N (%)			Sig.
	Q1	Q2	Q3		Q1	Q2	Q3		Q1	Q2	Q3	
Awaiting cannula removal	0 (0.0)	0 (0.0)	0 (0.0)	n/a	0 (0.0)	1 (3.9)	0 (0.0)	0.44	0 (0.0)	0 (0.0)	0 (0.0)	n/a
D/L or TTO	6 (20.0)	5 (25.0)	2 (9.1)	0.38	6 (35.3)	11 (42.3)	10 (40.0)	0.90	1 (8.3)	3 (23.1)	1 (8.3)	0.46
Awaiting transport	1 (3.3)	1 (5.0)	1 (4.5)	0.54	2 (11.8)	0 (0.0)	0 (0.0)	**0.045**	0 (0.0)	1 (7.7)	0 (0.0)	0.39
No delay	17 (56.7)	10 (50.0)	12 (54.5)	0.90	9 (52.9)	9 (33.6)	11 (44.0)	0.09	10 (83.3)	7 (53.9)	6 (50.0)	0.18
Ongoing OT/PT	3 (10.0)	4 (20.0)	5 (22.7)	0.43	0 (0.0)	5 (19.2)	3 (12.0)	**0.007**	0 (0.0)	1 (7.7)	3 (25.0)	0.13
Safeguarding	0 (0.0)	0 (0.0)	1 (4.5)	0.32	0 (0.0)	1 (3.9)	0 (0.0)	0.44	0 (0.0)	1 (7.7)	1 (8.3)	0.60
Awaiting placement	1 (3.3)	0 (0.0)	1 (4.5)	0.65	0 (0.0)	0 (0.0)	1 (4.0)	0.42	0 (0.0)	0 (0.0)	0 (0.0)	n/a
Awaiting district nurse	2 (6.7)	0 (0.0)	2 (9.1)	0.41	0 (0.0)	0 (0.0)	1 (4.0)	0.42	0 (0.0)	0 (0.0)	2 (16.7)	0.11

Results are expressed in N (%) where appropriate. Significance (*p* value), describing the comparison between JanuaryMarch 2023 (Q1), April–June 2023 (Q2) and July–September 2023 (Q3) for each hospital. Significance set at *p*<0.05. *p*-values in bold are significant. D/L = discharge letter; MFFD = medically fit for discharge; N = number; n/a = not applicable; OT = occupational therapy; PT = physiotherapy; Sig.= *p*-value; TTO = to take out.

### Trends in DKA outcomes following interventions that can reduce the length of stay post-DKA

There was no significant difference in DKA duration between the three time periods across all hospitals (median DKA duration (hours): Jan–Mar 2023 vs Apr–Jun 2023 vs July–Sept 2023: Hospital A 12.0 vs 11.2 vs 11.9 (*p*=0.87), Hospital B 18.8 vs 12.2 vs 17.4 (*p*=0.06), Hospital C 12.6 vs 16.2 vs 16.2 (*p*=0.61) (Table 4)). However, there was a significant increase in the length of stay in Hospital A despite the interventions (Hospital A 2.8 vs 4.8 vs 8.8 (*p*=0.05), Hospital B 5.0 vs 3.0 vs 6.0 (*p*=0.15), Hospital C 3.9 vs 4.2 vs 3.7 (*p*=0.80) (Table 4)). This was mainly influenced by the time taken by patients to become medically fit for discharge (time from DKA resolution to becoming MFFD: Hospital A 1.7 vs 3.2 vs 5.9 (*p*=0.02), Hospital B 3.6 vs 2.5 vs 4.7 (*p*=0.10), Hospital C 1.5 vs 1.6 vs 2.4 (*p*=0.50) (Table 4)).

**Table 4:** Differences in outcomes in Hospital A, B and C between January–March 2023 (Q1), April–June 2023 (Q2) and July–September 2023 (Q3).

Parameters	Hospital A median (IQR)			*p* value	Hospital B median (IQR)			P value	Hospital C median (IQR)			*p* value
	Q1	Q2	Q3		Q1	Q2	Q3		Q1	Q2	Q3	
DKA duration (hours)	12.0 (10.5–18.5)	11.2 (8.6–16.7)	11.9 (7.6–22.6)	*p*=0.87	18.8 (7.7–30.7)	12.2 (9.2–19.2)	17.4 (13.8–26.6)	p=0.06	12.6 (10.0–17.2)	16.2 (7.2–24.8)	16.2 (13.1–19.1)	*p*=0.61
LOS (days)	2.8 (2.0–6.3)	4.8 (3.8–12.4)	8.8 (2.4–13.1)	***p*=0.05**	5.0 (3.7–9.8)	3.0 (1.9–9.0)	6.0 (4.0–8.8)	p=0.15	3.9 (2.6–8.5)	4.2 (2.5–15.2)	3.7 (2.5–7.9)	*p*=0.80
Time from DKA resolution & MFFD (days)	1.7 (1.1–4.4)	3.2 (2.4–5.5)	5.9 (1.4–11.4)	***p*=0.02**	3.6 (2.7–7.5)	2.5 (0.7–5.7)	4.7 (2.3–7.4)	p=0.10	1.5 (0.9–1.7)	1.6 (0.9–1.7)	2.4 (1.4–6.0)	*p*=0.50
Time from MFFD to time of discharge (hours)	7.3 (4.1–10.6)	8.9 (3.4–14.4)	7.9 (3.3–10.5)	*p*=0.80	6.5 (4.0–9.2)	8.9 (6.3–35.3)	5.4 (4.0–7.9)	p=0.15	8.1 (5.3–26.0)	6.4 (3.8–51.9)	7.1 (5.7–18.5)	*p*=0.86
Time from DKA diagnosis to diabetes doctor review (hours)	11.7 (3.6–20.3)	15.7 (3.7–19.9)	17.8 (7.7–35.7)	*p*=0.19	10.6 (3.2–24.2)	6.2 (1.6–15.7)	14.2 (2.8–23.6)	p=0.25	5.4 (3.4–25.6)	11.5 (4.8–16.0)	7.4 (4.9–57.7)	*p*=0.92
Time from DKA diagnosis to DSN review (hours)	37.0 (19.9–75.9)	20.5 (11.0–48.4)	24.3 (20.3–65.6)	*p*=0.15	40.1 (22.2–51.7)	12.7 (2.9–26.9)	16.4 (11.7–24.2)	**p=0.01**	20.4 (19.4–33.5)	24.6 (19.8–36.9)	20.1 (16.3–20.9)	p=0.24
Reviewed by diabetes team N (%)	29 (97.0%)	20 (100.0%)	22 (100.0%)	***p*=0.50**	16 (95.0%)	26 (100.0%)	25 (100.0%)	p=0.22	12 (100.0%)	13 (100.0%)	12 (100.0%)	n/a

Results are expressed in median (IQR) where appropriate. Significance (p value), describing the comparison between January–March 2023, April–June 2023, and July–September 2023 for each hospital. Significance set at *p*<0.05. *p-*values in bold are significant.

DKA = diabetic ketoacidosis; DSN = diabetic specialist nurse; IQR = interquartile range; LOS = length of stay; MFFD = medically fit for discharge; N = number; n/a = not applicable.

## Discussion

Our dataset gives insight into various factors and their trends impacting the length of hospital stay following DKA episodes. Ongoing medical treatment for precipitating factors of DKA and optimising glycaemic control are the predominant factors influencing patients before they become medically fit for discharge in our cohort. Factors known to prolong the overall length of stay include DKA severity,^10^ underlying illness leading to DKA,^11^ new-onset diabetes^12^ and a higher Charlson Comorbidity Index.^6^^,^^7^

We did not see a significant reduction in the length of stay following DKA. Siddique *et al* found that interventions aimed at reducing hospital length of stay among socioeconomically disadvantaged individuals with DKA were inconclusive.^13^ Yong *et al* found no change in the mean length of stay post-implementation of an assisted discharge planning tool in 71 DKA patients in Christchurch Hospital, New Zealand.^14^ These studies and our study underscore the intricacies inherent in mitigating hospitalisation duration post-DKA and suggest that simple interventions may not yield substantial improvements in outcomes, emphasising the need for more comprehensive strategies to minimise resource utilisation effectively.

The National Institute of Health Research (NIHR) published an assessment of interventions to reduce the length of stay in 2014.^15^ Most studies examining the implementation of clinical care pathways reported positive impacts on patient outcomes, including length of stay. However, interventions targeted at discharge planning showed only small reductions in length of stay.

While we did not notice a significant improvement in the outcomes, our study has several strengths, adding new data to the scientific literature. We have developed and tested a data collection tool through expert consensus. This can be utilised in future studies, enabling data compilation and comparison between different settings. We have defined various factors that affect the length of stay post-DKA resolution, which can help in future study designs. Further, we reached a consensus through multidisciplinary team discussions advising that people admitted with DKA can be deemed medically fit for discharge if the person is clinically well, glucose is <20 mmol/L and ketones <0.6 mmol/L in two consecutive hours. This is important as we noticed that people are awaiting either glycaemic control or DSN review for a day or two after the DKA resolution. Adopting the policy can speed up discharge once appropriate follow-up is in place, thus reducing NHS bed pressure. It will also speed up the person to return to his usual daily living, thereby minimising deconditioning through hospital stay. However, we recommend at least one review by a diabetes team member during the hospital stay or a follow-up arranged as an outpatient to prevent readmission. We aim to evaluate the impact of this intervention in our centre and report in future studies as well, as with more time, we may see improvements in outcomes in future.

Our study has certain limitations. The short duration of follow-up after the third intervention may have resulted in the observed lack of significant improvements. While there was a variation in the number of DSNs across the three hospitals, these numbers were proportionate to each hospital's population. Nonetheless, future research should specifically examine whether this variation influences delays in care provision and consequently affects the length of stay. This QIP can be further developed in future by exploring the impact of comorbidities through the CCI, ethnicity, age, type of discharge (simple, complex, rapid^4^ and DKA readmissions. This will help develop more specific interventions, which may be more successful in reducing the overall length of stay.

## Conclusion

Our QIP sheds light on the complexities of reducing hospitalisation duration post-DKA and the limited impact of simple interventions. Further research and collaborative efforts are warranted to develop effective strategies to expedite discharge processes and enhance overall patient care in this clinical setting. Our comprehensive tracking and analysis tool can facilitate the optimisation of future intervention modalities.

## Funding

No funding was received.

## Declaration of generative AI in scientific writing

During the preparation of this work the author(s) used ChatGPT to improve language and readability. After using this tool/service, the author(s) reviewed and edited the content as needed and take(s) full responsibility for the content of the publication.

## CRediT authorship contribution statement

**Ankita Gupta:** Writing – review & editing, Writing – original draft, Visualization, Methodology, Investigation, Formal analysis, Data curation. **Benedict Brazier:** Writing – review & editing, Writing – original draft, Visualization, Validation, Project administration, Investigation, Data curation. **Lakshmi Rengarajan:** Writing – review & editing, Writing – original draft, Visualization, Validation, Project administration, Methodology, Investigation, Data curation. **Parth Narendran:** Writing – review & editing, Writing – original draft, Visualization, Project administration, Methodology, Investigation, Funding acquisition, Formal analysis, Conceptualization. **Punith Kempegowda:** Writing – review & editing, Writing – original draft, Visualization, Validation, Supervision, Software, Resources, Project administration, Methodology, Investigation, Formal analysis, Data curation, Conceptualization.

## Declaration of competing interests

The authors declare that they have no known competing financial interests or personal relationships that could have appeared to influence the work reported in this paper.

## Appendix Supplementary materials

Image, application 1

Image, application 2

Image, application 3

## References

[1] Gibb FW, Teoh WL, Graham J, Lockman KA (2016). Risk of death following admission to a UK hospital with diabetic ketoacidosis. Diabetologia.

[2] Kitabchi AE, Nyenwe EA (2006). Hyperglycemic crises in diabetes mellitus: diabetic ketoacidosis and hyperglycemic hyperosmolar state. Endocrinol Metab Clin North Am.

[3] Ooi E, Nash K, Rengarajan L (2021). Clinical and biochemical profile of 786 sequential episodes of diabetic ketoacidosis in adults with type 1 and type 2 diabetes mellitus. BMJ open diabetes Res care.

[4] Dhatariya KK (2022). The management of diabetic ketoacidosis in adults-An updated guideline from the Joint British Diabetes Society for Inpatient Care. Diabet Med.

[5] Kempegowda P, Coombs B, Nightingale P (2017). Regular and frequent feedback of specific clinical criteria delivers a sustained improvement in the management of diabetic ketoacidosis. Clin Med (Lond).

[6] Lee MH, Calder GL, Santamaria JD (2018). Diabetic ketoacidosis in adult patients: an audit of factors influencing time to normalisation of metabolic parameters. Intern Med J.

[7] Ata F, Khan AA, Khamees I (2023). Clinical and biochemical determinants of length of stay, readmission and recurrence in patients admitted with diabetic ketoacidosis. Ann Med.

[8] Association of British clinical diabetologists. Discharge planning for adults with diabetes 2022*.* https://abcd.care/sites/default/files/site_uploads/JBDS_Guidelines_Archive/JBDS_10_Discharge_Planning_Guideline_January_2022_Archive.pdf. [Accessed March 2, 2024]

[9] Rengarajan LN, Nash K, Ooi E, Cooper C, Birchenough A, Owen M (2022). Sustaining improvement in diabetes-related ketoacidosis management through a quality improvement projects. Br J Diabetes.

[10] Guisado-Vasco P, Cano-Megías M, Carrasco-de la Fuente M (2015). Clinical features, mortality, hospital admission, and length of stay of a cohort of adult patients with diabetic ketoacidosis attending the emergency room of a tertiary hospital in Spain. Endocrinol Nutr.

[11] Freire AX, Umpierrez GE, Afessa B (2002). Predictors of intensive care unit and hospital length of stay in diabetic ketoacidosis. J Crit Care.

[12] Ata F, Khan AA, Khamees I (2023). Differential evolution of diabetic ketoacidosis in adults with pre-existent versus newly diagnosed type 1 and type 2 diabetes mellitus. BMC Endocr Disord.

[13] Siddique SM, Tipton K, Leas B (2021). Interventions to reduce hospital length of stay in high-risk populations: a systematic review. JAMA Netw open.

[14] Yong KW, Moore M, Lunt H (2014). Medically facilitated discharge of adult diabetic ketoacidosis admissions: precipitants and average length of stay. N Z Med J.

[15] Miani C, Ball S, Pitchforth E (2014).

