# Supplementary material for: Determining factors influencing hospital stay for individuals admitted with diabetes-related ketoacidosis – findings from DEKODE length of stay quality improvement project
Source: Clin Med (Lond). 2024 Oct 19;24(6):100255. doi: 10.1016/j.clinme.2024.100255 (PMC11564021; doi:10.1016/j.clinme.2024.100255)
Supplement: Supplementary file 2 [file mmc2.docx]

**Supplementary material 2: Data collection tool**

| **Question** | **Answer options** |
| --- | --- |
| Which Hospital was the patient admitted to? | - A - B - C |
| Year of birth | Day/month/year |
| Gender | - Male - Female |
| Ethnicity | - White – British - White – Irish - Other white background - Black or Black British-Caribbean - Black or Black British-African - Other Black Background - Asian or Asian British-Indian - Asian or Asian British-Pakistani - Asian or Asian British-Bangladeshi - Chinese - Other Asian Background - Mixed – White and Black Caribbean - Mixed – White and Black-African - Other Mixed Background - Other Ethnic background - Not Known |
| Charlson Comorbidity Index (CCI) prior to hospitalisation |  |
| Admission date and time | Day/month/year |
| Did the patient die during this episode | - Yes - No |
| Date and time of discharge | Day/month/year |
| Date and time of DKA diagnosis | Day/month/year |
| Date and time of DKA resolution | Day/month/year |
| Date and time when patient became medically fit for discharge | Day/month/year |
| Reason for hospital stay from DKA resolution until medically fit for discharge | - DSN review for optimising anti-diabetes medicines and education - Glycaemic control- on variable rate intravenous insulin infusion - Glycaemic control- blood glucose not in safe range to be MFFD - Ongoing investigations and treatment - Medical specialist review including diabetes doctor review - Surgical specialist review - Psychiatric team review - Allied Health professional review - Other: insert free text |
| Reason for hospital stay after becoming medically for discharge | - Discharge letter or TTO awaited for more than 3 working hours after MFFD (Monday to Friday 0800 to 1700 excluding bank holidays) - Awaiting transport - Ongoing OT/PT - Safeguarding - Awaiting placement - Awaiting District nurse - No delay - Other: insert free text |
| Did the patient have a diabetes doctor review during this episode? | - Yes - No |
| If yes:  Date and time when patient was first seen by a doctor from diabetes team during this DKA episode | Day/month/year |
| Did the patient have diabetes specialist nurse review during this episode? | - Yes - No |
| If yes:  Date and time when patient was first seen by a DSN from diabetes team during this DKA episode | Day/month/year |

DKA = diabetic ketoacidosis; DSN = diabetes specialist nurse; MFFD = medically fit for discharge; OT = occupational therapy; PT = physiotherapy; TTO = to take out
