# Supplementary material for: Determining factors influencing hospital stay for individuals admitted with diabetes-related ketoacidosis – findings from DEKODE length of stay quality improvement project
Source: Clin Med (Lond). 2024 Oct 19;24(6):100255. doi: 10.1016/j.clinme.2024.100255 (PMC11564021; doi:10.1016/j.clinme.2024.100255)
Supplement: Supplementary file 3 [file mmc3.docx]

**DEKODE Working Group**

| Name | Affiliation | Email |
| --- | --- | --- |
| Saima Kauser-Malik | University Hospitals Birmingham NHS Trust | [Saima.Kauser-Malik@nhs.net](mailto:Saima.Kauser-Malik@nhs.net) |
| Anu Ann Abraham | University Hospitals Birmingham NHS Trust | [anuabraham7@gmail.com](mailto:anuabraham7@gmail.com), |
| Muhammad Ali Karamat | University Hospitals Birmingham NHS Trust | muhammad.karamat@uhb.nhs.uk |
| Sanjay Saraf | University Hospitals Birmingham NHS Trust | [Sanjay.Saraf@uhb.nhs.uk](mailto:Sanjay.Saraf@uhb.nhs.uk) |
| Nevil Philip | UHB University Hospitals Birmingham NHS Trust | [nevilc.philip@nhs.net](mailto:nevilc.philip@nhs.net) |
| Ragavendran Govindaraj Sureshkumar | UHB University Hospitals Birmingham NHS Trust | [r.sureshkumar1@nhs.net](mailto:r.sureshkumar1@nhs.net) |
| Manbir Duggal | UHB University Hospitals Birmingham NHS Trust | [manbirsduggal@gmail.com](mailto:manbirsduggal@gmail.com) |
| Shamanth Soghal | UHB University Hospitals Birmingham NHS Trust | [ShamanthSoghal@gmail.com](mailto:ShamanthSoghal@gmail.com) |
| Dineshwaran Rajendran | UHB University Hospitals Birmingham NHS Trust | dineshwaran25@gmail.com |
| Pranav Viswanath Iyer | Birmingham Medical School, University of Birmingham | [pxv164@student.bham.ac.uk](mailto:pxv164@student.bham.ac.uk) |
| Wai Nga Alice Yip | Birmingham Medical School, University of Birmingham | [aliceyip16@hotmail.co.uk](mailto:aliceyip16@hotmail.co.uk) |
| Meghnaa Hebbar | Birmingham Medical School, University of Birmingham | [meghna.hebbar@gmail.com](mailto:meghna.hebbar@gmail.com) |
| Francesca Pang | Birmingham Medical School, University of Birmingham | [FHP849@student.bham.ac.uk](mailto:FHP849@student.bham.ac.uk) |
| Anjitha Anilkumar | Birmingham Medical School, University of Birmingham | [anjitha14@outlook.com](mailto:anjitha14@outlook.com) |
| Carina Synn Cuen Pan | Birmingham Medical School, University of Birmingham | [18pancarina@gmail.com](mailto:18pancarina@gmail.com) |
